# Molecular signatures from omics data: From chaos to consensus

**DOI:** 10.1002/biot.201100305

**Published:** 2012-04-23

**Authors:** Jaeyun Sung, Yuliang Wang, Sriram Chandrasekaran, Daniela M Witten, Nathan D Price

**Affiliations:** 1 Institute for Systems BiologySeattle, WA, USA; 2 Department of Chemical and Biomolecular Engineering, University of IllinoisUrbana, IL, USA; 3 Center for Biophysics and Computational Biology, University of IllinoisUrbana, IL, USA; 4 Department of Biostatistics, University of WashingtonSeattle, WA, USA

**Keywords:** Diagnostics, Disease classification, Systems biology, Translational bioinformatics

## Abstract

In the past 15 years, new “omics” technologies have made it possible to obtain high-resolution molecular snapshots of organisms, tissues, and even individual cells at various disease states and experimental conditions. It is hoped that these developments will usher in a new era of personalized medicine in which an individual's molecular measurements are used to diagnose disease, guide therapy, and perform other tasks more accurately and effectively than is possible using standard approaches. There now exists a vast literature of reported “molecular signatures”. However, despite some notable exceptions, many of these signatures have suffered from limited reproducibility in independent datasets, insufficient sensitivity or specificity to meet clinical needs, or other challenges. In this paper, we discuss the process of molecular signature discovery on the basis of omics data. In particular, we highlight potential pitfalls in the discovery process, as well as strategies that can be used to increase the odds of successful discovery. Despite the difficulties that have plagued the field of molecular signature discovery, we remain optimistic about the potential to harness the vast amounts of available omics data in order to substantially impact clinical practice.

The identification of molecular signatures from omics data has many promising applications including omics-based tests for disease-specific diagnostics and accurate phenotype classification. This is however, plagued by issues with data reproducibility – this review discusses the potential pitfalls in the discovery process and strategies for overcoming these issues in order to achieve personalized medicine.

## 1 Introduction

**Figure 1:**
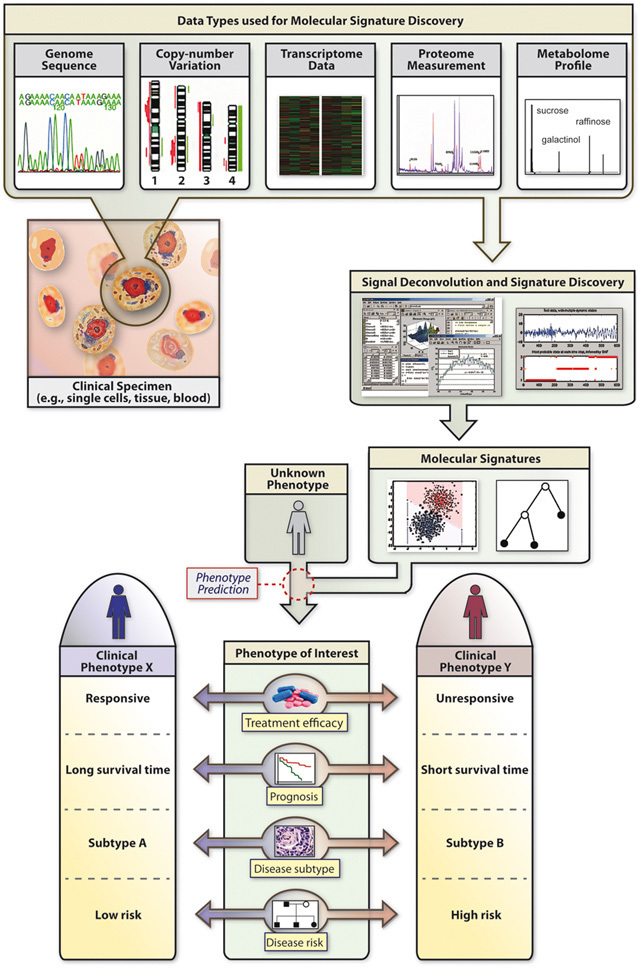
Overview of the discovery and application of molecular signatures from omics data. Molecular signatures can be derived from a broad range of omics data types (e.g. DNA sequence, mRNA, and protein expression) and can be used to predict various clinical phenotypes (e.g. response to therapy, prognosis) for previously unseen patient specimens.

In recent years, new high-throughput measurement technologies for biomolecules such as DNA, RNA, and proteins have enabled unprecedented views of biological systems at the molecular level. The fields of research associated with obtaining and understanding such measurements – for instance, genomics, transcriptomics, and proteomics – are sometimes referred to in aggregate as *omics*. Given molecular measurements taken from a biological system, a natural goal is to develop a statistical model that uses these measurements to predict a clinical outcome of interest, such as disease status, survival time, or response to therapy. In this paper, we will discuss the process of using omics data to discover a *molecular signature*. Here, we define a molecular signature as *a set of biomolecular features (e.g. DNA sequence, DNA copy number, RNA, protein, and metabolite expression) together with a predefined computational procedure that applies those features to predict a phenotype of clinical interest on a previously unseen patient sample.* A signature can be based on a single data type [1–4] or on multiple data types [5–8]. The overall process of identifying molecular signatures from various omics data types for a number of clinical applications is summarized in Fig. 1.

Many possible clinical phenotypes might be predicted by a molecular signature; a few examples include prediction of disease risk and progression [9–11], response to therapeutic drugs [12–14] and their physiological toxicity [15, 16], and time to disease recurrence or death [17, 18]. A successful case of the clinical utility of omics-derived molecular signatures is MammaPrint [19], a diagnostic test approved by the Food and Drug Administration for clinical use. MammaPrint is a 70-gene expression signature used to predict breast cancer prognosis and to determine the appropriate therapeutic regimen for lymph node negative breast cancer patients with either ER positive or negative. The list of 70 genes was selected based on correlation with clinical outcome (distant metastasis vs. no metastasis), and underwent successful validations on independent patient cohorts [20, 21].

Despite a few notable exceptions such as MammaPrint, the successful discovery of molecular signatures has largely been hampered by limited reproducibility and variable performance on independent test sets [22–28], as well as difficulty in identifying signatures that outperform standard clinical measurements like the cardiovascular disease risk C-reactive protein (CRP) [29]. These difficulties can be attributed in large part to the low S/N inherent to omics datasets, the prevalence of batch effects in omics data, and molecular heterogeneity between samples and within populations [30]. These issues are exacerbated by the fact that the datasets used to develop molecular signatures tend to have small sample sizes relative to the number of molecular measurements [31]. Moreover, improper study design, inconsistent experimental techniques, and flawed data analysis can lead to further challenges in the process of molecular signature discovery. Though there has been marked progress in the field of molecular signature discovery in recent years, there remains a clear need for further improvements in the discovery process in order for omics-based technologies to begin to achieve their full clinical potential.

## 2 The four stages of molecular signature discovery

Roughly speaking, the process of molecular signature discovery on the basis of omics data consists of four major stages:

(i) Defining the scientific and clinical context for the molecular signature;

(ii) Procuring the data;

(iii) Performing feature selection and model building; and

(iv) Evaluating the molecular signature on independent datasets.

In the sections that follow, we will discuss each of these stages in turn.

### 2.1 Stage 1: Defining the scientific and clinical context

We first consider the problem of selecting a suitable omics data type for a molecular signature. A signature intended to distinguish between cancer and normal tissue could be based upon a number of omics data types; for instance, one might base the signature upon gene expression measurements, if it is believed that this type of cancer shows altered expression of some genes relative to normal tissue, or upon DNA sequence data, if samples from this cancer are characterized by particular mutations or copy number changes. However, given a clinical phenotype of interest, certain types of omics data might not form the basis for a sensible molecular signature. For instance, it would not be reasonable to attempt to create a molecular signature to screen for adult onset (type II) diabetes on the basis of DNA sequence data alone because an individual's DNA sequence remains essentially static throughout his or her lifetime, but risk of developing the disease may change.

We now consider the clinical context of the molecular signature. A gene expression-based signature that can distinguish between cancer and normal tissues would be of little practical use if a physician can easily make the same distinction using standard (and less expensive) clinical approaches. Similarly, a signature that can distinguish between two subtypes of cancer is useful only if those two subtypes differ in some clinically relevant way, such as in survival time or response to therapy, since otherwise the information about cancer subtype provided by the molecular signature may not serve a practical purpose. As an example, gastrointestinal stromal tumors (GISTs) and leiomyosarcomas (LMSs) are remarkably similar morphologically and were originally classified as being the same cancer. However, it was found that they respond very differently to distinct therapies, and thus a signature that can distinguish between these two diseases based on gene expression in tissue samples can be useful [3]. An example outside of cancer involves the use of metabolomic information from human serum to noninvasively diagnose and monitor Alzheimer's disease (AD) progression [32–34].

### 2.2 Stage 2: Data procurement

The development of a molecular signature requires the availability of adequate omics data for which the clinical phenotype of interest is available. In general, there are two ways in which such data can be procured: new data can be collected experimentally for the specific purpose of molecular signature discovery, or else existing data (collected previously for other purposes, and generally publicly available) can be used. There are pros and cons of either approach. Collecting new data has a major advantage, in that all aspects of the experiment can be carefully controlled. On the other hand, data collection is expensive, and given the large sample sizes necessary for successful molecular signature discovery, using existing datasets may be a more feasible approach. There are a number of public data repositories from which omics data and associated clinical phenotypes can be obtained. For instance, a useful source of gene expression data is NCBI Gene Expression Omnibus (GEO), a repository of over 26000 studies that continues to grow at a rapid pace. Other public data repositories include ArrayExpress [35] and Sequence Read Archive [36]. Regardless of how the data are procured, it is crucial that the samples correspond to the scientific and clinical context of interest, as described in the previous section.

In order for a dataset to be suitable for molecular signature discovery, the samples must be collected under appropriate experimental and analytical conditions. As an example, any biological factors (such as gender, age, or ethnicity) that may be associated with the clinical phenotype of interest or with the omics measurements should be taken into consideration in the process of data procurement. In addition, to reduce the prevalence of *batch effects*, factors such as sample collection and processing procedures, laboratory personnel, study run-dates, reagent sources, measurement instruments, and data processing methods should be carefully controlled [37–39]. Deviations in these protocols can have a surprisingly large effect on the omics measurements obtained, often larger than the effect of the clinical phenotype of interest [40]. Ideally, there should be no association between the clinical phenotype of interest and these factors. For instance, in the case of a molecular signature that classifies tissue samples into tumor versus normal, there should be no difference between the tumor and normal samples in terms of the laboratory personnel who performed the sample preparation, or the sample run-dates. If experimental and analytical procedures are not carefully controlled, they can result in confounding with the clinical phenotype of interest, leading to the development of a classifier that performs very well on the data used in its development, but that will perform poorly on independent test samples.

**Figure 2:**
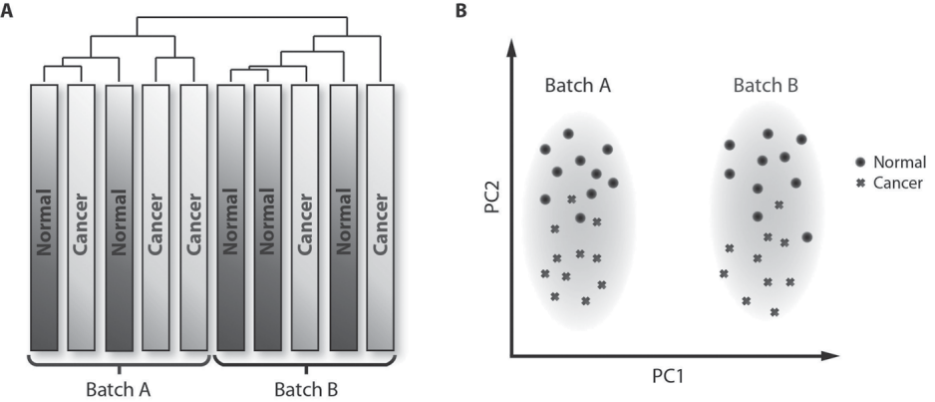
Two hypothetical scenarios in which (A) hierarchical clustering and (B) principal components analysis reveal that covariates other than the clinical outcome of interest have resulted in considerable discrepancies between patient populations. Here, batch characteristics and not group labels (cancer versus normal clinical specimens) are responsible for most of the observed variation among the samples. Such batch effects can arise due to changes in experimental protocols, data-processing techniques, or laboratory personnel at any point in the experimental process.

To the extent that analytical and experimental factors do vary among the samples, these factors should be explicitly included in the model used to develop the classifier. Normalization procedures have been proposed that are intended to reduce the effect of measured and unmeasured external factors on omics data [41]; however, good experimental design remains the best strategy [42]. Exploratory data analysis techniques, such as hierarchical clustering (Fig. 2A) and principal components analysis (Fig. 2B) can be useful tools to assess the extent to which covariates that are not of primary interest may have affected the data.

When existing data is used for omics-based molecular signature discovery, it is particularly important that sufficient information about the experiment is available to ensure that good experimental design was followed (this will be discussed further in Section 4). For instance, if the run date for each sample is not given, then one cannot be certain that the clinical phenotype of interest is not highly confounded with run date.

Unfortunately, many omics studies have sample sizes substantially smaller than would be required for the successful identification of molecular signatures. A molecular signature that is developed on the basis of a small number of samples is more likely to be sensitive to technical and biological sources of noise and variation, and less likely to capture the aspects of the data that are truly associated with the phenotype of interest. This exacerbates the risk of over-fitting, wherein the signature performs well on the samples used for signature development but fails to correctly predict the clinical phenotype of interest in previously unseen samples. In contrast, global molecular characteristics of a particular phenotype may become more apparent as sample size increases. Therefore, having a large sample size, while by no means a cure-all, will greatly improve the odds that a given attempt at molecular signature discovery will prove fruitful. Integrating across multiple datasets of the same phenotypes from different labs can also help to amplify the primary biological signal of interest relative to noise. Of course, whether a given sample size is “large” or “small” depends the type of omics data being used for signature discovery, the clinical phenotype of interest, and many other factors.

### 2.3 Stage 3: Feature selection and model building

Once a scientific and clinical context has been established and one or more datasets have been identified, we can develop a molecular signature through (i) feature selection; and (ii) model building. These two tasks can be performed together or separately.

We first consider the task of feature selection. A typical omics experiment simultaneously measures thousands or even millions of biological features (e.g. single nucleotide polymorphisms, RNA transcripts, protein levels) on each patient sample. However, just because thousands of molecular measurements are obtained does not mean that thousands of molecular measurements should be used in the molecular signature. Since financial cost, technical practicality, and measurement robustness are important criteria to select signatures, then if all else is equal, a signature that could be ultimately measured via PCR or Western blot is favored over a signature that requires a technique involving many more protocol steps, such as in omics measurements. In order to reduce the number of features used in molecular signature development, *feature selection* is performed. Feature selection can be performed in a *supervised* manner (e.g. the 20% of features that are most associated with the clinical phenotype of interest are selected), or in an *unsupervised* manner (e.g. the 20% of features with the highest variance are selected). Once a set of features has been selected, only those features are used in the model building process, which is described next.

We now consider the task of *model building* – i.e. the process of developing a specific computational procedure that can be applied to the omics measurements from a future patient sample in order to predict the unknown clinical phenotype of interest for that sample. There are many possible approaches to building such a model, and in particular, the type of model used will depend on the clinical phenotype of interest. For instance, if we wish to develop a molecular signature to predict time to cancer recurrence, then a Cox proportional hazards model might be appropriate. On the other hand, to develop a molecular signature that can distinguish between cancer and normal tissue, one could use a classification approach, such as logistic regression, support vector machines, neural networks, or linear discriminant analysis. Some approaches for model-building involve first performing an unsupervised technique, such as clustering or principal components analysis, followed by a supervised procedure, such as logistic regression.

Once we have developed a model, how can we determine whether it is any good? Despite certain drawbacks [43, 44], the most popular approach for evaluating model performance in this context is *cross-validation*. (Cross-validation is also often used for tuning parameter selection, though that application is outside of the scope of this paper.) Cross-validation involves repeatedly splitting the samples in the dataset into training and test sets, performing all aspects of feature selection and model building on the training set, and evaluating the model's performance on the test set. Cross-validation can also be used to select from among a small number of possible models: the model with the smallest cross-validation error rate should be chosen.

Cross-validation is a simple and intuitive approach to estimating the error rate associated with a model, but it must be performed with care. Most importantly, within each cross-validation fold, no information about the test set can be used in building the model on the training set. For instance, suppose that one performs feature selection by selecting the 10% of features whose *t*-statistics between cases and controls are largest. One then performs logistic regression, using only these features, to develop a classifier to distinguish between cases and controls. How should the cross-validation error rate be calculated? Consider the following two approaches:

Approach 1 (incorrect): identify the 10% of features that differ most between cases and controls, and use only those features henceforth. Perform cross-validation by repeatedly splitting the samples into training and test sets, fitting a logistic regression model on the training set (using just the 10% of features previously identified), and then evaluating the model's performance on the test set.

Approach 2 (correct): perform cross-validation by repeatedly splitting the samples into a training set and a test set. Within each training set, identify the 10% of features that differ most between cases and controls, and use those features to fit a logistic regression model. Then, evaluate the performance of this model on the test set.

The difference may seem subtle, but it is in fact crucial. Approach 1 will yield a woeful underestimate of the true error rate, because the 10% of features that differ most between cases and controls were identified using all of the samples, including those in the test set, rather than simply the training samples. In effect, if Approach 1 for cross-validation is taken, then perfect error rates can potentially be obtained even on datasets in which the “case” and “control” labels were assigned randomly! On the other hand, in Approach 2, feature selection is performed using the training set within each cross-validation fold, and so the resulting cross-validation error rate is valid. Unfortunately, the difference between Approaches 1 and 2 is often overlooked, and the literature is rife with papers in which extraordinarily low, but grossly inaccurate, cross-validation error rates are reported because some variant of Approach 1 has been performed. The key principle is that in computing cross-validation error rates, within each cross-validation fold only training observations can be used in any aspect of feature selection or model development. Deviations from this principle, even if seemingly innocuous, may result in dramatic underestimates of error.

At the end of the feature selection and model building process, the molecular signature must be *locked down* – i.e. the precise computational procedure used to convert a new omics sample into a prediction of the clinical phenotype must be completely specified. Only then can the molecular signature be fairly evaluated on independent datasets, as described next.

### 2.4 Stage 4: Evaluation on independent datasets

Once a promising molecular signature has been identified, its performance needs to be evaluated on completely independent patient samples. Unlike cross-validation, wherein the test set is drawn from the same population as that of the training set, an *independent* sample is one that is completely separate from the set of samples used for feature selection and model building. In particular, this means that the test set is *not* simply a random split from a large dataset (even if sequestered and not used in any training sets). If a molecular signature performs well on a truly independent set of samples, then this provides evidence that it will likely generalize to future patient samples. However, the amount of evidence for a molecular signature's performance based on independent data depends critically upon specific characteristics of the independent dataset.

*Lower level of evidence. Good performance on an independent dataset collected at the same institution using carefully controlled protocols*. This provides evidence that the molecular signature works well in this particular setting, with these protocols, with the patient profile at this institution, etc. However, it may not hold up elsewhere. At the very least, its ability to work in other settings has not been demonstrated.

*Higher level of evidence. Good performance on multiple independent datasets collected at multiple institutions*. Success in this setting is the best evidence that a molecular signature will perform well on future patient samples. This indicates that the signature is robust to the kinds of things that might change between locations: namely, aspects of the biology of the populations that tend to go to particular hospital, sample preparation and measurement techniques used, and so forth.

Evaluation of a molecular signature on fully independent patient samples is the gold standard for assessing its performance. Unfortunately, it often is the case that molecular signatures that seem promising in the feature selection and model building stage (i.e. that have very low cross-validation error rates) exhibit poor performance on independent data.

## 3 Disclosing all experimental protocols, datasets, and source code

A key principle of science is that other researchers must be able to reproduce the results. In order for a molecular signature to be reproduced, three essential pieces of information are required: (i) the experimental and analytical protocols; (ii) the raw data; and (iii) the source code used to develop the signature. We discuss each of these points in turn.

In order for a molecular signature to be fully understood by other researchers, detailed information on the experimental protocol, including the patient selection criteria and experimental and analytic procedures, must be made available. Without this information, one cannot determine the scientific or clinical contexts in which the molecular signature is intended, appropriate, or useful.

Second, in order for a molecular signature to be reproduced, the omics data used in its development, as well as the associated metadata and clinical data, must be made available. If the data are not released, then it simply is not possible for other research groups to determine whether the molecular signature is valid.

Finally, even if the data are made available, other research groups will not be able re-derive the molecular signature based on the same data used for its discovery, and confirm that the signature does truly work well on independent data, unless all data processing techniques and all analytical and computational methods are made available. Unfortunately, in practice this information often is not provided in sufficient detail. For instance, there is a tendency for authors to publish a list of the features (e.g. genes) involved in the signature, without the detailed mathematical formulas required to understand precisely how the omics measurements are used in order to predict the clinical phenotype of interest. This is a major obstacle to progress in the field, as other research groups cannot reproduce or validate – much less build upon – research that is not sufficiently reported. In order to address this problem, the source code used to develop the molecular signature should be released. Ideally, this code should encompass all aspects of signature development, from processing and normalization of the raw omics data, to feature selection to model building to evaluation on an independent dataset.

## 4 Using multiple datasets for molecular signature discovery

Thus far, we have described the development of a molecular signature on the basis of a single dataset, followed by evaluation of the signature on one or more independent datasets. However, in principle, multiple datasets can be used for molecular signature discovery. In fact, this can often lead to more accurate and more broadly applicable molecular signatures.

When a molecular signature is developed on the basis of a single dataset and then tested on an independent dataset, its performance tends to degrade severely in the independent dataset relative to its cross-validation error rate in the dataset used for development. This drop in performance can stem from heterogeneity between studies due to underlying variance in the biology of the patients studied, as well as from technical variations in measurement, normalization, and analysis. That is, a signature developed using a single dataset may overfit certain aspects of the dataset that are not of primary scientific interest, leading to poor performance on independent data. This problem can be partially overcome by developing the signature on the basis of multiple datasets, collected at different institutions and at different time points [45–47]. (However, the primary clinical phenotype of interest, such as tumor versus normal, must be balanced between the datasets in order to avoid confounding between the datasets and the clinical phenotype.)

## 5 Using multiple data types for molecular signature discovery

**Figure 3:**
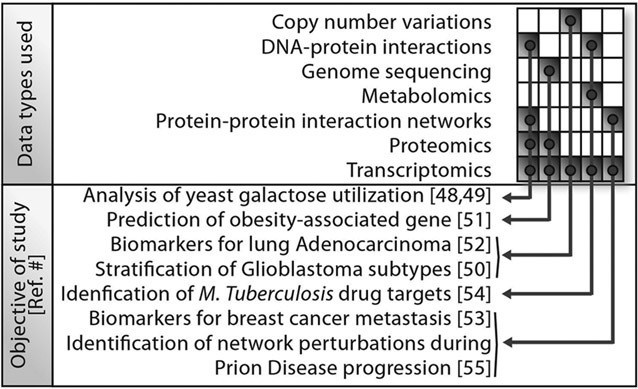
Combining different types of data across different measurement platforms can lead to more accurate molecular signatures for characterizing or predicting clinical phenotypes. Rows and columns of the checkered box correspond to data types and published studies, respectively. The collection of gray boxes in each column represents the combination of data types used in a particular study. The arrows designate the objective of each study.

Given the complexity of biological systems in general and pathological processes in particular, there is an upper limit to how well a molecular signature developed on the basis of a single data type (e.g. genome-wide expression on DNA microarrays) can predict disease phenotypes and clinical outcomes. Integrating multiple types of omics data may allow for the development of increasingly accurate and robust molecular signatures. For example, gene expression data can be combined with copy number variation data or DNA sequence data. Successful multi-scale integration of different types of biological information is one of the current challenges in systems biology [48, 49]. In Fig. 3, we provide brief summaries of a few recently published studies [48–55] in which multiple data types were used for molecular signature discovery.

A number of methods to combine diverse types of omics data across different measurement platforms and laboratories have been proposed [48, 49, 56], in order to more accurately select clinically relevant features or to develop better molecular signatures. For example, English and Butte evaluated data from 49 obesity-related studies that used different experiment types, including DNA microarrays, genome-wide association, proteomics, and RNAi knockdowns [51]. The investigators found that the biomolecules reported to be associated with obesity in individual studies had little overlap with previously known obesity-related genes. The investigators then determined a gene to be obesity-related if five or more studies reported the gene to be obesity-related. Using this approach of feature selection, they were able to identify a higher proportion of known obesity related genes than from any of the 49 individual studies, and also discovered new genes for which there was compelling support of association with obesity [51]. This demonstrated that even straightforward integration of multiple omics data types can substantially improve the feature selection process. In a study by Lu et al. [52], the investigators integrated data types in order to perform more effective feature selection: they identified 475 genes that were differentially expressed between lung adenocarcinoma and normal tissue, and that were also located in copy number varying regions. This gene set was used to create a predictive model for patient survival, which was then shown to be accurate on three independent patient cohorts. Advances in integrating diverse omics data types may lead to a reduction in spurious signal caused by technical limitations of individual platforms, and an increased ability to identify molecular signatures associated with the underlying mechanistic roles in disease pathogenesis.

## 6 A network-based approach to molecular signature discovery

The use of network-based approaches is a promising avenue for molecular signature discovery. These networks represent a complex web of interactions among diverse components in a cell, and can be used to develop more reproducible and accurate molecular signatures by exploiting the underlying biology of the system. Network-based approaches extend beyond simple integration of different omics data types, and can involve evaluating complex interactions that can vary due to disease or other perturbations.

Most statistical methods for feature selection and model building do not take a network-based approach: they implicitly assume that the features are independent, or that they are only weakly dependent, though this has begun to change in recent years [57–59]. However, in most biological contexts, the assumption of independent features is certainly violated. For instance, genes regulated by the same set of transcription factors, or genes encoding enzymes for the same metabolic pathway, will tend to show correlated expression. Therefore, rather than treating each feature in an omics dataset individually, it may be preferable to map from the high-dimensional molecular space to a much smaller number of (possibly curated) functional biological networks. Mapping features into functional sets reduces dimensionality, increases the statistical power to detect small but coordinated disease perturbations, and improves the interpretability of the resulting molecular signatures.

In order to identify features that are associated with a clinical phenotype of interest, features can be mapped onto a priori defined and manually curated modules or “pathways”. Gene Set Enrichment Analysis (GSEA) [60] is a very widely used approach to investigate pathway-level changes in gene expression data, and more recent proposals have also been made. One recently developed approach to identifying pathway-based molecular signatures for phenotype classification is the Differential Rank Conservation (DIRAC) method [61]. Unlike GSEA or other enrichment methods that usually return *p*-values for gene set enrichment, DIRAC builds a network-based molecular signature that identifies robust differences in pathway activity between two disease states.

However, one major caveat to such pathway-based approaches is that a priori defined pathways do not fully represent the complexity of the underlying biology, and may not be accurate within the particular physiological context. To overcome this limitation, molecular features can be mapped into more comprehensive interaction networks, such as protein-protein or protein-DNA interaction networks, which can be much more comprehensive and unbiased, as well as disease and context specific. Specifically, biological networks can be used as a structured framework to integrate omics data for the purpose of molecular signature development. For example, Chuang et al. [53] integrated microarray gene expression data with protein–protein interaction networks to identify network-based prognostic biomarkers for breast cancer metastasis, and generated novel hypotheses regarding cancer progression. The average sub-network activity, defined in this study as a function of expression levels of genes that compose the sub-network, was used to predict clinical outcome of breast cancer specimens. The network-based markers displayed better predictive accuracy on an independent dataset than markers selected without network information. In another study, Nibbe et al. [62] used proteins that were differentially expressed between normal and cancer colon tissue from proteomics experiments as seeds to identify sub-networks enriched in these differentially expressed proteins from the human protein interaction network. Then, the mRNA expression profiles of the components of these sub-networks were used as input features to a support vector machine in order to classify colorectal cancer and normal samples. The prevalence of these networks being perturbed in colon cancer was demonstrated by these features alone being sufficient to achieve 90% classification accuracy in independent validations.

In the particular case of prion disease, a set of neurodegenerative disorders caused by the misfolding of prion proteins in the brain, Hwang et al. [55] analyzed the dynamic network perturbations during the onset and progression of disease. In this study, infectious prion proteins were delivered into the brains of living mice, and were harbored within the tissue for different time-spans of disease progression. At the end of each time-point, gene expression measurements were taken from harvested diseased brain tissue, and subsequently mapped onto physical protein interaction networks for comparative analysis. Intriguingly, this study showed reproducible perturbations that occurred in core networks that could be monitored prior to the manifestation of disease symptoms.

In the work summarized above, thousands of feature measurements for static biological states were used to characterize molecular networks. However, a more complete understanding of molecular networks requires perturbing the biological system under study in order to understand how the network components, as well as the clinical phenotype of interest, are affected by those perturbations. For example, stimulating one or more signaling pathways using in vitro cytokine assays can lead to different immunologic and metabolic responses in different diagnostic phenotypes [63], such as different disease progression levels. In a study by Hale et al. [64], the investigators used a cocktail of cytokines and mitogens to stimulate whole blood cells from patients with different stages of systemic lupus erythematosus, an autoimmune disease. They then used flow cytometry to measure multiple signaling responses at the single-cell level, generating a highly multiplexed view of intracellular signaling network activity during disease progression. They found that robust changes in signaling protein interactions in response to stimuli were good indicators of disease stage. Therefore, evaluating cell response after an activating stimulus may serve as a compelling approach for incorporating perturbations into patient classification going forward.

## 7 Are my features truly correct?

Given that two molecular signatures seem to perform well on independent datasets, how can we decide which is better? If all else is equal, we should prefer the molecular signature for which there is a plausible biological mechanism, as such a signature is much more likely to hold up in future patient samples as opposed to having overfit the data used in its development. Ideally, if sufficient numbers of samples were available, then a molecular signature's performance on one or many independent datasets would be the preferred way of assessing its suitability, regardless of whether or not a mechanism for its performance is known. But in reality, sample sizes are limited, and thus a molecular signature for which there is a plausible biological mechanism tends to be more convincing than one for which no such mechanism is known. Such biologically motivated signatures can also hold great promise to be developed as companion diagnostics for therapies, which may be motivated by the underlying mechanism. Thus, while lack of a known biological mechanism underlying a molecular signature certainly does not preclude its use provided that it works well in practice on independent samples, mechanistic information can increase our confidence that the signature will hold up to further scrutiny.

## 8 Pervasive bias in reported results

Another major challenge in omics-based molecular signature discovery is the prevalence of overly optimistic accuracies in reported results. This problem is not unique to omics research but is problematic in many data-driven research settings [65]. Such bias can occur for a number of reasons: (i) research groups tend to report only the best results among many attempted approaches; and (ii) only positive results are published. Consequently, across the literature there is an overly optimistic view of how well molecular signatures perform. This pervasive bias is not necessarily the result of faulty science in any particular lab, but rather is a consequence of the way in which science is conducted and reported. This is responsible, in part, for the fact that many reported molecular signatures have not held up in follow-up studies.

## 9 Conclusions

In this paper, we have discussed some of the key considerations and challenges facing the discovery of omics-based molecular signatures of clinical phenotypes, such as good experimental design, careful data procurement, avoidance of over-fitting, validation on independent datasets, and integration of multiple datasets and data types. For guidance to the reader, Box 1 summarizes the key steps in molecular signature discovery that were discussed throughout this paper. We hope that this methodological checklist will aid investigators interested in identifying omics-based molecular signatures.

Since the emergence of the field of omics-based molecular signature discovery, researchers have developed an improved understanding of how to discover (and how not to discover!) such signatures. The field is still young, and as time passes, best practices in this area will continue to evolve. Currently, the number of validated and useful molecular signatures is disappointingly (but not surprisingly) small relative to the number of signatures that have been reported in the literature. However, we remain optimistic that as experimental and analytical practices improve, as sample sizes increase, and as techniques for data type integration continue to develop, omics-based molecular signatures will indeed transform the practice of medicine.

**Box 1. Steps for the development of molecular signatures on the basis of omics data**

**Step 1. Establishing the scientific and clinical contex**

- Clearly define clinical phenotypes of interest
- Ensure that, if discovered, a molecular signature has the potential to be useful in the clinic
- Only use types of omics data that are suitable for addressing the task of interest
- Determine acceptable sensitivity and specificity

**Step 2. Collecting omics data for molecular signature discovery**

When collecting new experimental data, ensure that:

- sufficient sample size can be obtained
- all aspects of the experimental and analytical procedures are carefully controlled to avoid batch effects
- no confounding occurs between datasets of different phenotypes from factors unrelated to phenotype of interest

>

When using existing data, ensure that:

- sufficient sample size can be obtained
- sufficient patient information is available for omics samples
- proper normalization is implemented to make samples comparable across different datasets

Consider integrating multiple datasets and data types:

- approach with caution
- can lead to molecular signatures that are more accurate and robust

**Step 3. Developing molecular signatures through feature selection and model building**

- Perform feature selection in either a supervised or an unsupervised manner
- Choose models that are well-suited for the context of the study and nature of phenotypes of interest
- Consider mapping features onto biological pathways or more comprehensive interaction networks
- Consider choosing models that show clear insight into plausible biological mechanisms
- Ensure that all cross-validation steps are performed correctly
- Approach favorable cross-validation results with caution

**Step 4. Evaluating performance on independent datasets**

- Test promising molecular signatures on independent datasets
- Independent test sets are not created equal. The strength of evidence from an independent test is based on the characteristics of the independent dataset used (i.e. evaluating on data from multiple, different sites is a more stringent test than evaluating on data from only the same institution)

**Step 5. Disclosing information on all aspect of study to enhance reproducibility**

- Encourage the evaluation of the molecular signature by independent research groups
- Disclose: information on the clinical context in which molecular signature is intended, patient selection criteria, clinical data (i.e. patient information), raw data, meta-data (if applicable), data processing and normalization methods, feature selection and model building methods, experimental protocols, records on study run-dates, lab technicians, reagent sources, etc., analytical methods, and source code

**Step 6. Reporting all performance results to mitigate bias in public literature**

- Encourage the objective assessment of molecular signatures by reporting both positive and negative outcomes (i.e. correct and incorrect predictions, respectively)
- Make data publicly available after publication

## Biographical Information

**Nathan Price** is Associate Professor at the Institute for Systems Biology in Seattle, WA. He is also Affiliate Faculty at the University of Washington and at the University of Illinois, Urbana-Champaign. He serves on the Board of Directors and Scientific Advisory Board for the P4 Medicine Institute. His work has been recognized by an NIH Howard Temin Pathway to Independence Award in Cancer Research, *Genome Technology's* “Tomorrow's PIs, ” an NSF CAREER award, a Roy J. Carver Young Investigator Award, and the Camille-Dreyfus Teacher-Scholar Award.

## Biographical Information

**Jaeyun Sung** is currently a Ph.D. candidate in the Department of Chemical and Biomolecular Engineering at the University of Illinois, Urbana-Champaign. His research interests lie in the field of computational and systems biology for personalized medicine. For his doctoral work, he is developing novel bioinformatics approaches to identify various kinds of molecular signatures from genomic information of human disease specimens (e.g. tumor biopsies). Using these molecular signatures, he aims to elucidate the causal perturbations within complex, intracellular biomolecular networks during disease manifestation and progression.
